# Incidence and predictors of treatment failure among children on first-line antiretroviral therapy in Amhara Region Referral Hospitals, northwest Ethiopia 2018: A retrospective study

**DOI:** 10.1371/journal.pone.0215300

**Published:** 2019-05-01

**Authors:** Birtukan Aklog Yihun, Getiye Dejenu Kibret, Cheru Tesema Leshargie

**Affiliations:** 1 College of Health Science, Debre Markos University, Debre Marko, Ethiopia; 2 Debre Markos Referral Hospital, Debre Marko, Ethiopia; Sefako Makgatho Health Sciences University, SOUTH AFRICA

## Abstract

**Background:**

Human immunodeficiency virus (HIV) infection is a major public health concern globally, especially in sub-Saharan African countries. Even though determining the incidence of treatment failure and its predictor is a crucial step to reduce the problem, there is limited information indicating the incidence and predictors of treatment failure among children in Ethiopia. Therefore, this study was conducted to assess the incidence and predictors of treatment failure among children on first-line antiretroviral therapy (ART) in Amhara Region referral hospitals, Northwest Ethiopia.

**Methods:**

An institution-based retrospective follow-up study was conducted from January 30, 2011, to January 30, 2018. A total of 402 children on first-line antiretroviral therapy were selected with a simple random sampling method in Amhara Region Referral Hospitals, Northwest Ethiopia. Data were extracted by reviewing patients’ ART intake and follow-up forms using pretested and structured checklists. The collected data were entered into Epidata Version 4.2 and analysis was done using STATA Version 13. Bivariable and multivariable Cox proportional hazards regression models were fitted to identify predictors of treatment failure.

**Results:**

A total of 402 records of children on antiretroviral therapy (ART) were reviewed and treatment failures rate within the follow-up period were 12.19% (95% CI: 8.5, 15.88). This study also found that the overall incidence density rate was 3.77% per 100 person-years observation. Virologic failure accounts 48.98% followed by immunologic (28.57%) and mixed failures (22.44%). Poor ART adherence (AHR: 4.6, 95%CI: 1.61, 13.20), drug regimens, AZT-3TC-NVP (AHR: 5.2, 95%CI: 1.9, 14.26), and AZT-3TC-EFV (AHR: 6.26, 95% CI: 1.88, 20.87), Children whose both parent were died (AHR: 2.8, 95%CI: 1.07, 7.37) and world health organization (WHO) clinical stage-4 (AHR: 2.95, 95%CI: 1.04, 8.366) were found to be predictors for treatment failure among children.

**Conclusion:**

The proportion of treatment failure among children on first-line ART in Amhara Region referral hospitals, Northwest Ethiopia was found to be high. Nearly half of the children experienced Virologic failure. Poor ART adherence, children whose parents`died without parents, WHO clinical stage-4 at baseline and type of regimen patients took were found to be predictors of first-line ART treatment failure. Therefore, expanding access to routine viral load, CD4 and clinical monitoring is mandatory to detect and early intervene of treatment failures’ to improve outcomes for children on ART. Patient caregivers or parents should strictly support children on medication adherence. Training to health professionals should be given time-based on revised guidelines, and follow up of treatment outcome should be monitored nationally to take the appropriate intervention.

## Introduction

Acquired immune deficiency syndrome (AIDS) is a viral infection caused by the human immunodeficiency virus (HIV) that weakens the immune system and makes the body susceptible to secondary and opportunistic infections [1]. The disease continues to be a major global health priority, where more than 2 million children worldwide are infected with HIV, approximately 90% of whom live in sub-Saharan Africa. Sub-Saharan Africa has the highest burden of HIV/AIDS worldwide [2–4]. It remains the most heavily affected region, accounting for 71% of all new HIV infections and an estimated 430,000 new HIV infections occurred among children under the age of 15 [4]. Antiretroviral therapy (ART) coverage rose from 7% in 2003 to 42% in 2008, with especially high coverage achieved in eastern and southern Africa (48%) [2, 3, 5].

AIDS develops very rapidly among Infants and young children living with HIV, have a high risk of poor outcomes, with up to 52% of children born with HIV dying before the age of 2 years and one third before year one in the absence of any intervention [6]. There were 120,000 children who died of AIDS-related causes in 2016[4]. A serious challenge for children with HIV is maintaining long-term adherence to treatment regimens, and thus virological suppression and prevention of treatment failure [7]. The long duration of therapy needed for HIV-infected children requires maximal efficacy, minimal toxicity, and prevention of development of drug resistance which requires consideration of ways to minimize the occurrence of resistance and treatment failure [8]. There is a growing problem of treatment failure Even if many HIV-positive clients accessed ART and not a common diagnosis in most centers especially in low-middle income countries including Ethiopia in which there is a delay in detecting it and switching to second-line treatment, which results in an increased rate of mortality [6, 9].

Evidences showed that different predictors affect treatment failure. The most common predictors are age [7, 10–14], gender [10–12], being orphan [15], time from ART initiation [15], poor adherence to ART failure [10, 11, 16–18] [19], nevirapine (NVP)-based regimen [13, 20], drug side-effects, drug toxicity [10, 11, 16, 17], nutritional status [11], pretreatment CD4 count [1, 10, 11, 13, 14, 16, 19, 21], WHO clinical stage [9–11, 16, 19, 21] and tuberculosis co-infection [9, 19]. A previous study [22] does not address important predictor like repeated measurement for CD4 which this includes.

To reduce the problem of HIV/AIDS, ART was endorsed in 2003 and in January 2005, free ART was launched in Ethiopia [10, 23]. However, according to 2016, around 21,686 children under the age of 15 were taking ARV. ART coverage is still low and only 33% under-15 years old age children are receiving the treatment [reference]. In addition, recently viral load monitoring was accessed in some centers of Ethiopia [6].

With the existence of this intervention, the burden of HIV/AIDS still continued to be high, and the national HIV prevalence is 1.16% in [specify the year here] [10]. Therefore, this study aimed to determine the incidence and predictors of treatment failure among children on first-line antiretroviral therapy in Amhara region referral hospitals, Northwest Ethiopia from January 30, 2011, to January 30, 2018. The finding encourage clinicians to provide targeted treatment approach which provides appropriate treatment care and follow up for HIV infected children and optimizes treatment outcomes, through decreasing the duration children stay on a failing regimen and the resulting drug resistance, potentially decreasing morbidity and mortality.

## Methods and materials

### Study design, area and period

An institution-based retrospective study was conducted to assess treatment failure and its predictors at Debre Markos and Felege Hiwot Referral hospitals in Amhara region, from 2011 to 2018. The health facilities were selected from five referral hospitals found in the region. Debre Markos Referral Hospital (DMRH) is found in Debre Markos town, Amhara national regional state Northwest Ethiopia. The town is located 299 Km northwest of Addis Ababa, the capital city of Ethiopia. The hospital provides service for an estimated of 2,397,876 total catchment populations. It started providing ART service with aid from the Regional Health Bureau and the Ministry of Health in April 2005 [24, 25]. Felege Hiwot Referral Hospital is one of the government-sponsored ART centers at Bahir Dar town; the capital of Amhara Regional State. It is located approximately 565 km northwest of Addis Ababa [26]. The hospital also provides services to patients from surrounding rural villages and nearby towns. The two hospitals provide voluntary counseling and testing, prevention of mother to child transmission (PMTCT), antiretroviral therapy (ART) and treatment of opportunistic infection services [25]. There were a total of 239 and 352 children who ever enrolled to ART at Debre Markos and Felege Hiwot referral Hospitals, respectively.

#### Population

All HIV infected children who were under 15 years of age, who initiated ART between January 30, 2011 and January 2018, and who took first line ART for at least six months were included in this study.’ Those children with incomplete medical record for important predictors were excluded from the study.

#### Sample size determination and sampling procedure

The sample size was calculated by using the formula for survival analysis [27], $\text{E}=\frac{{\left({\text{Z}}_{\alpha /2}+{\text{Z}}_{\beta }\right)}^{2}}{\text{P}\left(1-\text{P}\right){\left(\text{lnHR}\right)}^{2}}$, Where, Alpha (α) = 0.05, Beta (β) = 0.2, HR- Hazard ratio, E- Number of event, P = cumulative occurrence of treatment failure, N (sample size) = E / P (E), where P (E) = probability of event, Used reference for sample size calculation [11]. The total sample size was 443 (10% added to account for lost or incomplete documents), with 95% CI, 80% power. After determining the number of case and checking for completeness and uniformity of record keeping for each year at each hospital, a fraction of the total sample size proportional to total caseload seen by each hospital during the given time interval was allocated. Then, simple random sampling was applied to select all (190 from Debre Markos Referral Hospital and 212 from Bahir Dar Referral Hospital) individual client record’s using their medical registration number used in the registration process.

### Operational definitions

**Children:** individuals with age less than 15 years old [28].
**Event:** Any types of treatment failure after initiation of first line ART (clinical, Immunological and virological failure.
**Treatment failure:** is categorized as clinical, immunological and virological failure and defined as follows [6, 10].
**Clinical failure:** New or recurrent clinical event indicating advanced or severe immune deficiency (WHO clinical stage 3 and 4 clinical condition with exception of TB) after 6 months of effective treatment.
**Immunological failure:** Persistent (at least 2 CD4 measurements) CD4 levels below 200cells/mm for children younger than 5 years and, CD4 levels below 100 cells/mm for older than 5 years.
**Virological failure:** Viral load above 1000 copies/mL based on two consecutive viral load measurements in 3 months, with adherence support following the first viral load test.
**Censored:** Individuals other than treatment failure (individual like lost from follow up, dead, transferred out and still on first line ART at the end of follow up).
**Adherence:** The extent to which a client’s behavior coincides with the prescribed regimen as agreed upon through a shared decision making process between the client and the health care provider[6], evidenced from follow up card. A Combination of tools is used to assess patient medication adherence. Based on the remaining Pill count clinician’s can consider good, fair and poor adherence and it can perform better when combined with self-reported adherence. We consider good, fair and poor adherence if the percentage of missed dose is <95%, 85–94% and < 85% respectively.

### Data collection instruments

A structured English version checklist was developed and used for data extraction from the patients’ medical records on the Federal Ministry of Health Antiretroviral therapy (ART) follow up form and pediatrics HIV intake form. These records included; clients’ age, gender, weight, height, duration of follow up, clinical and laboratory data (WHO stage, opportunistic infections, CD4 count and white blood cell count), the ART regimen and prophylaxis (Co-trimoxazole or Isoniazid), and adherence on a structured data retrieval form. Trained health professionals (nurses) working in the ART clinic were recruited as data collectors and supervisors.

### Data collection procedures and quality assurance

One day training was given for data collectors on data extraction system, data collection tools and objectives of the study. To assure the quality of data, the data collection checklist was pretested. After the pretest, necessary modification of data collection tool was made. Strict follow-up and supervision were carried out during data collection by principal investigators and feedback were given on a daily bases. The collected data was reviewed and checked for completeness before data entry.

### Data processing and analysis

The collected data were entered using EpiData version 4.2 statistical software and exported to Stata version 13 statistical software for further analysis. The Kaplan Meier survival curve methods were used to estimate the time to treatment failure. The Log-rank test was used to compare survival curves between different categories of explanatory variables. The Cox proportional-hazards model was used to assess the predictors associated with treatment failure. To see the effect size of predictors on time to treatment failure, bivariable and multivariable Cox-proportional hazards regression model was fitted. Variables having p-value ≤ 0.25 in the bivariable analysis were fitted into multivariable analysis. Hazard Ratios (HR) with 95% confidence intervals was computed and a statistical significance was declared at 5% level (p<0.05). The necessary assumptions for Cox proportional hazard model were checked using Schoenfeld residuals test and graphically with log-log Cox adjusted survival estimate. The model fitness was checked using the Nelson- Aalen cumulative hazard rate relative to Cox-Snell residuals.

### Ethical considerations

Ethical clearance was obtained from Ethical review committee of Debre Markos University Health Science College. The ethics committee formally waived to extract patient data to address objectives aimed by this study. Then, permission was also obtained from the administrative bodies of each hospital (Debre Markos referral and Felege Hiwot referral hospitals) to access the ART client’s database and charts. Confidentiality was also maintained by not recording the identity that describes the individual and limiting the access of the data only for concerned bodies like principal investigator and advisors.

## Results

### Socio-demographic characteristics

After excluding 41 HIV positive children records due to incompleteness, we reviewed a total of 402 HIV positive children charts that were registered from January 30, 2011, to January 29, 2018. During the data extraction process, 41 HIV positive children medical record charts were excluded due to incomplete documentation and lack of important variables. Out of the included participants, slightly more than half, 225 (55.97%) were male and the mean age was 6.15 (SD = ± 3.48) years. One hundred ninety (47.26%) and 212 (52.74%) had a follow up at Debre Markos and Felege Hiwot Referral Hospital respectively. The majority, 329 (81.84%) of study participants were from the urban area and the mean age of caregivers was 37.58 (SD = ± 7.17) years. Most, 332(82.59%) of the children had a relationship with their parents as a caregiver. slightly more than half, 219 (54.48%) of both the mother and father of children were alive. Less than one third, 113 (28.83%) of the care giver were a government employee, and 242 (61.89%) were married (Table 1).

**Table 1:** Socio-demographic characteristics of children on ART at Northwest Ethiopia referral hospitals, 2011to 2018 (N = 402).

Socio-demographic characteristics		Frequency(N)	Percent (%)
**Age categories of under five children**	<1	19	4.73
	1 to 4	124	30.85
	5–15	259	64.42
**Sex**	Male	225	55.97
	Female	177	44.03
**Residence**	Urban	329	81.84
	Rural	73	18.16
**Parent status**	Both alive	219	54.48
	Either dead	122	30.35
	Both dead	36	8.95
	Unknown	25	6.22
**Age of care giver (years)**	20–40	233	57.96
	>40	169	42.04
**Relationship of care giver for the child**	Parent	332	82.59
	Relatives	45	11.19
	Guardians/neighbors	17	4.23
	Orphanage	8	1.99
**Occupation of care giver**	Government employee	113	28.83
	Private employee	19	4.85
	Merchant	84	21.43
	daily laborer	82	20.92
	Driver	10	2.55
	Farmer	55	14.03
	House wife	29	7.4
**Marital Status of Care Giver**	Single	10	2.56
	Married	242	61.89
	Divorced	43	11.00
	Widowed	96	24.55

### Baseline clinical and immunological factors

Clinically, one third, 129 (32.58%) of the study participants were categorized to WHO clinical stage 3 and the median CD4 cell count was 410cells (IQR 249–780). During ART follow-up, slightly more than two-thirds, 278 (69.33%) of children experienced opportunistic infection and the commonest, 71 (25.72%), was diarrhea. Most, 106 (72.1%) of the under-five children had appropriate developmental status while the majority, 222 (88.10%) of HIV positive children functional status were ambulatory when they start ART (Table 2).

**Table 2:** Baseline clinical and immunological characteristics of children on first line ART at Northwest Ethiopia referral hospitals, 2011to 2018 (N = 402).

	Frequency(N)	Percent (%)
**WHO Clinical stage**		
**Stage I**	129	32.58
**Stage II**	109	27.52
**Stage III**	129	32.58
**Stage IV**	29	7.32
**CD4 Count at initiation (cells/mm3)**		
**<50 mm^3^**	14	3.48
**≥ 50 mm^3^**	388	96.52
**Opportunistic infection at baseline**		
**Diarrhea**	71	25.73
**Tuberculosis**	48	17.39
**Meningitis**	7	2.55
**Pneumonia**	63	22.84
**Candidiasis**	21	7. 61
**URTIs**	10	3.62
**Skin disorders**	51	18.45
**Other**	5	1.81
**Baseline developmental status for <5 years children**		
**Appropriate**	106	72.11
**delayed**	39	26.53
**regression**	2	1.36
**Baseline functional status for ≥5 years children**		
**working**	17	6.74
**ambulatory**	222	88.10
**bed ridden**	13	5.16

### Medical and clinical follow up history of participants

One hundred forty (34.91%) of study participants were found on AZT-3TC-NVP ART regimen during ART initiation, and the majority, 362 (90.05%) of them had good ART adherence during their last follow-up period. Nearly one-third (32.8%) of the study participants had experience of regimen change during their ART follow-up time. Among the study participants, 49 (12.19%) had experienced ART side effects during their follow-up period, and mostly with AZT-3TC-NVP regimen, anemia being the commonest 15 (30.61%). Toxicity or side effects were the commonest 47 (35.6%) reason for regimen change. program shift from D4T to AZT or TDF, stock out of drugs and treatment failure were other reasons. During the follow–up time, majority 374 (93.27%) of the study participants did not take ART-prophylaxis, following birth from HIV infected mothers (PMTCT). Among the children on ART, 381 (94.78%) took Cotrimoxazole and 245 (61.25%) took on their follow-up time Isoniazid prophylaxis. The median ART clinic follow up period of participants was 35 months (IQR 18–62) months with minimum 6 months and maximum 84 months of follow-up, and nearly half, 205 (50.99%) of the participants were followed for less than 36 months. The majority, 334 (83.08%) of study participants were on ART follow-up at the time of the study, 8 (1.99%) were reported as lost, 10 (2.49%) dead, and 50 (12.44%) were transferred out (Table 3).

**Table 3:** Follow up data on factors related to ART and other medications children on first line ART at Northwest Ethiopia referral hospitals, 2011to 2018 (N = 402).

Variables	Frequency	Percentage (%)
**ART regimens given at first**		
D4t-3TC-NVP	65	16.21
D4t-3TC-EFV	18	4.49
AZT-3TC-NVP	140	34.91
AZT-3TC-EFV	82	20.45
TDF-3TC-EFV	28	6.98
AZT-3TC-LPV/R	38	9.45
ABC-3TC- LPV/R	24	5.97
Other	7	1.74
**ART adherence(last 3 months)**		
good	362	90.05
fair	14	3.48
poor	26	6.47
**Drug side effect**		
yes	49	12.19
no	353	87.81
**Regimen change/ substitution**		
yes	132	32.84
no	270	67.16
**ARV prophylaxis for PMTCT**		
given	27	6.73
not given	374	93.27
**Cotrimoxazole prophylaxis**		
given	381	94.78
not given	21	5.22
**Isoniazid prophylaxis**		
given	245	61.25
not given	155	38.75
**Month on ART**		
<36	205	50.99
36–60	89	22.14
≥ 60	108	26.87
**ART drug side effect**		
Yes	49	12.19
No	353	87.81
**Side effects**		
nausea	12	24.49
fatigue	2	4.08
headache	11	22.45
rash	6	12.25
anemia	15	30.61
Others	3	6.12
**Current status**		
Alive	334	83.08
Dead	10	2.49
Lost to follow up	8	1.99
Transfer out	50	12.44
**First line ART treatment failure**		
Yes	49	12.19
No	353	87.81
**Type of treatment failure**		
Virologic	24	48.98
Immunologic	14	28.57
Mixed(clinical+ immunologic)	4	8.16
Mixed (immunologic+virologic)	3	6.12
Mixed (clinical + Virologic)	3	6.12
Mixed (All)	1	2.04

### Incidence of treatment failure

**Fig 1:**
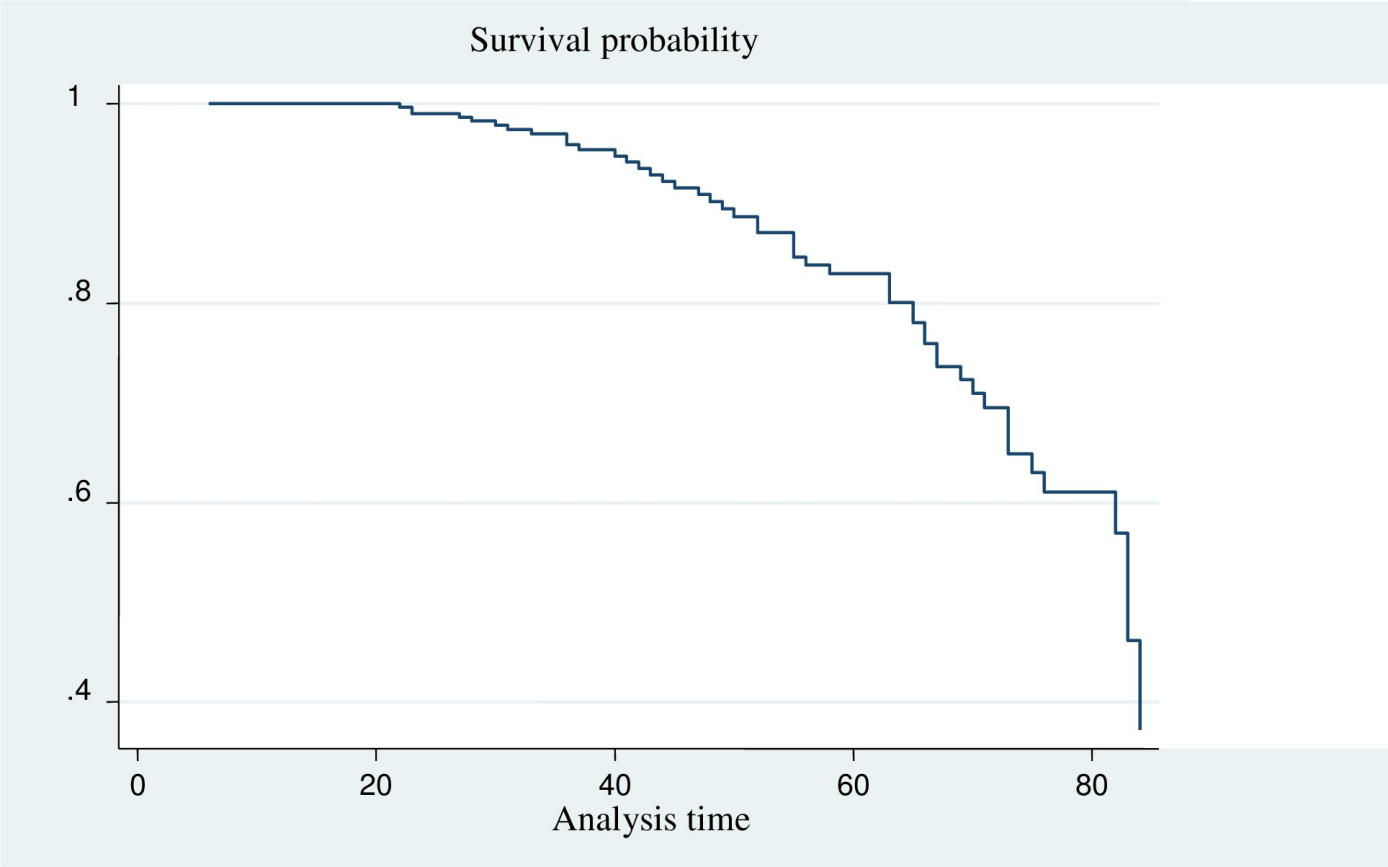
The survival probability of HIV positive children on ART at Debre Markos and Felege Hiwot referral hospitals from 2011 to 2018.

**Fig 2:**
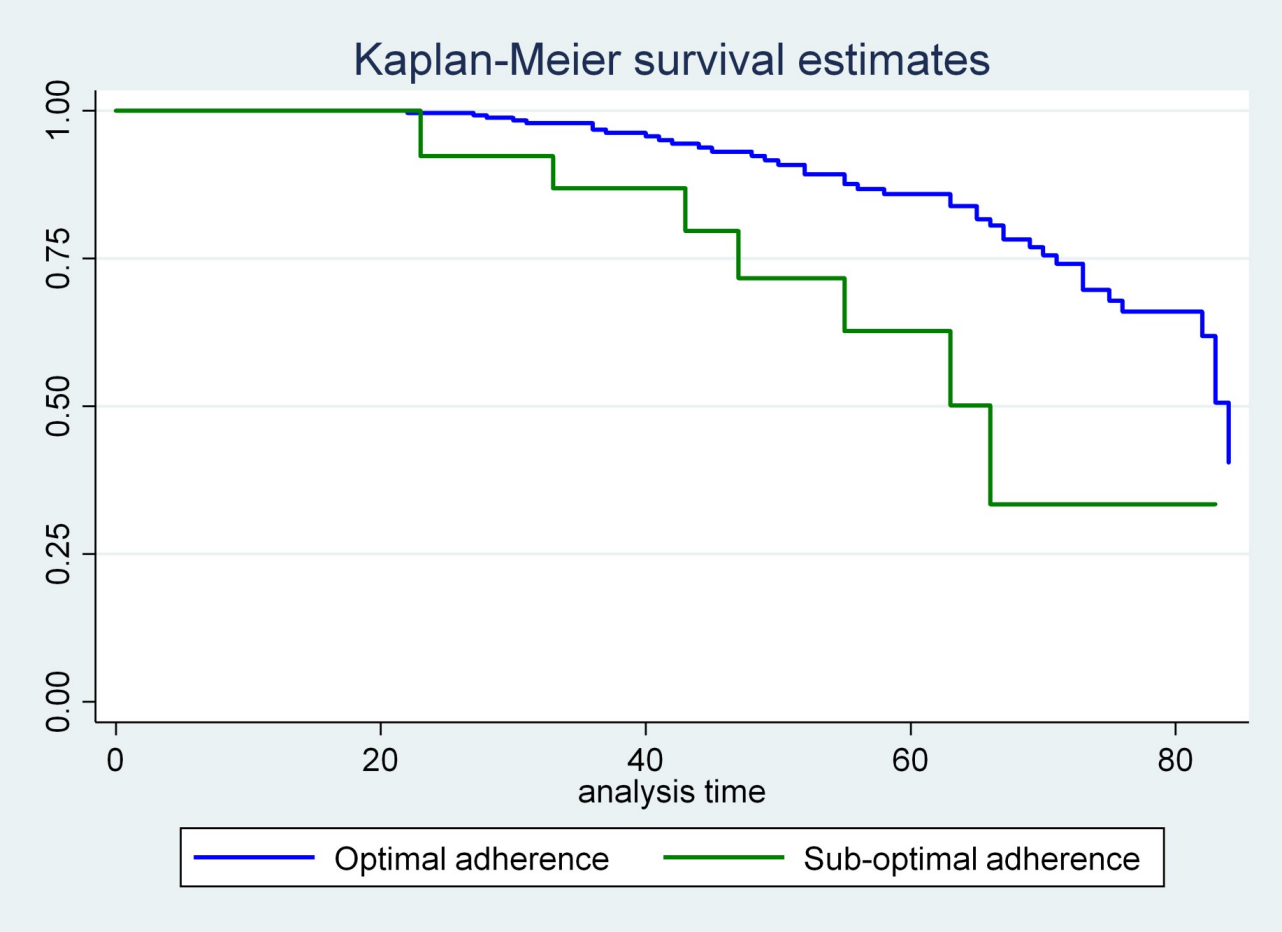
The survivorship of HIV positive children on ART who had optimal and suboptimal adherence during their follow up time at Debre Markos and Felege Hiwot referral hospitals from 2011 to 2018.

All study participants (402) who were followed for different periods in 7 years, a minimum of 6 months and a maximum of 84 months. The median survival time was 83 months with 1298.32 person-years follow up time. Forty-nine, 12.19% (95% CI: 8.5, 15.88) of participants experienced treatment failure. Of this, 48.98%, 28.57% and 8.16% of the children have experienced a Virologic, Immunologic and mixed (that means clinical+ immunologic) respectively. In addition, a small proportion, 6.12%, 6.12% and 2.04% of them were mixed (clinical + Virologic) and mixed (clinical, virological and immunological) respectively. The overall incidence density rate (IDR) was 3.77 (95%CI, 2.85, 4.99) per 100 person-years of observations (Figs 1 and 2).

### Bivariable and multivariable Cox proportional hazard analysis

In the Bivariable analysis age at initiation of ART, caregiver’s occupation, baseline WHO clinical stage, baseline CD4 status, residence, parent status, marital status of the caregiver, type of regimen, duration on ART, adherence, Isoniazid prophylaxis, ART side effects and regimen change or substitution were found significant predictors for treatment failure.

On multivariable analysis, children whose both parents died were 2.8 (AHR: 2.8, 95%CI: 1.07, 7.37) times at a higher risk of treatment failure as compared with those whose both parents alive. In addition, children who were presented with WHO clinical stage 4 was 2.95 (AHR: 2.95, 95%CI: 1.04, 8.366) times at a higher risk of experiencing treatment failure than those who were on WHO clinical stage 1 and 2. Moreover, children who had poor ART adherence were 4.6 (AHR: 4.6, 95% CI: 1.61, 13.20) times at a higher risk of experiencing treatment failure than the counterpart. In addition, children who are on AZT-3TC-NVP treatment regimen were 5.2 (AHR: 95%CI: 1.9, 14.26) times at a higher risk of treatment failure when compared with those who were on d4t-3TC-NVP. Being on AZT-3TC-EFV were also 6.26 (AHR: 95% CI: 1.88, 20.87) times at higher risk of treatment failure as compared with those who were on d4t-3TC-NVP (Table 4). Treatment failure result with d4T-3TC-NVP was associated with; it’s decreased usage on the research period, after phasing out. Patients on this regimen initially and who stayed till the revised guideline were changed to other regimens. So those patients were not followed for a long time on this drug.

**Table 4:** Predictors of treatment failure among children who are on ART at Northwest Ethiopia referral hospitals, 2011 to 2018 (N = 402), (using cox proportional regression).

	Event	Censored	P- value	
**Adherence status**				
Good	41	321	1.0	1.0
Fair	2	12 0.294	1.71(0.41, 7.13)	2.29(0.495,10.60)
Poor	6	20 0.004	5.94(2.43, 14.49)	4.61(1.61, 13.21)
**Regimen**				
d4t-3TC-NVP	9	56	1.0	1.0
d4t-3TC-EFV	3	15 0.079	0.78(0.21, 2.9)	3.99(0.88, 18.08)
AZT-3TC-NVP	21	119 0.001	2.09(.956, 4.58)	5.197(1.89,14.26)
AZT-3TC-EFV	10	72 0.003	3.85(1.55, 9.58)	6.26(1.88, 20.87)
TDF-3TC-EFV	2	26 0.069	11.26(2.24, 56.67)	5.53(0.84, 36.53)
AZT-3TC-LPV/R	8	30 0.056	0.64(.24, 1.73)	0.19 (0.14, 1.49)
ABC-3TC-LPR/R	4	20 0.067	0.72(0.20, 2.54)	0.02 (0.004, 0.59)
Others	1	6 0.103	1.70(.21, 13.84)	0.68 (0.13, 3.72)
**Parent status**				
Both alive	16	203	1.0	1.0
Either dead	19	103 0.135	1.72(0.87, 3.42)	2.50(.71, 8.76)
Both dead	10	26 0.035	2.96(1.34, 6.54)	2.82(1.27, 7.37)
Unknown	4	21 0.554	1.08(0.35, 3.28)	1.59(0.36, 7.02)
**WHO stage**				
WHO stage I & II	15	223	1.0	1.0
WHO stage III	26	106 0.177	1.05 (0.54, 2.04)	3.3(0.13, 9.65)
WHO stage IV	8	24 0.028	3.11(1.29, 7.47)	2.95(1.14, 8.37)

## Discussion

While antiretroviral therapy (ART) service is available worldwide, acquired immune deficiency syndrome (AIDS) was continuing to be a major global health priority problems especially in developing countries including Ethiopia [2, 3, 5]. Treatment failure is one of the factors which determine the effectiveness of ART intervention [7]. Therefore, this institution-based retrospective cohort study sought to estimate the incidence and predictors of treatment failure among HIV positive children on first-line ART in Northwest Ethiopia.

This study found that treatment failure among children was 12.19% (95% CI: 8.5, 15.88) with 3.77 per 100 per-year incidence rates. The current finding is consistent with the finding reported at Jimma University Hospital (11.5%) [21] and at four ART center hospitals Addis Ababa (14.4%) [11].

On the other hand, the incidence of treatment failure is found to be lower as compared with study findings reported by Gondar University Hospital (18.2%) [29], Fiche and Kuyu hospital Oromia region (18.9%) [16], Tanzania (57%) [20], Ghana (29%) [30], Uganda and Mozambique 29% [9]. In this study, virologic failure is the commonest type of treatment failure followed by immunologic failure and only a small proportion having a mixed failure, which is in line with a cohort study done in Ghana and Tanzania [20, 30]. However, immunologic and clinical failures were predominant in several studies conducted in Ethiopia [11, 16, 21].

The current study also identified the predictor variable for treatment failure and found that AZT-3TC-NVP and AZT-3TC-EFV regimen, suboptimal adherence, advanced WHO clinical staging and dead parents of children were predictors of treatment failure.

The current study found that children whose parents (both) died were predictors well the treatment failure. This is similar to a study conducted at Cambodia in HIV-Positive Children revealed that [10, 15]. In this current study, the existence of poor ART adherence among children on first-line ART was identified as a predictor variable for treatment failure supported by finding shown at Ugandan [18] and Fiche and Kuyu hospital Oromia region [16]. HIV positive children without parents or appropriate caregiver may have psychological depression or impairment and may not have good adherence which can be resulted in developing ART drug resistance with a run out of immune cells which end up in treatment failure. This implies tracing of a patient from ART clinic need to be strengthened using proper appointment calendar utilization, frequent updating of patient contact address to decreasing lost patients.

AZT based regimen was found as a predictor for treatment failure by the current which is similar to a study done in Addis Ababa [11]. In contrary, Nevirapine (NVP)-based regimen was identified as a predictor for virological failure as revealed on a study in Botswana [31], Mozambique and Uganda [9] and Tanzania [20], also in Thailand Non-nucleoside reverse transcriptase inhibitor type was one predictor of Virologic failure [12]. These differences may mirror clinicians’ preference in first-line treatment choice. Country-specific differences may potentially confound the relationships seen between combined ART regimen and treatment failure.

Similar to the current study advanced WHO clinical stage at baseline was a significant predictor of treatment failure, as studies show at Gondar University Hospital [29], Jimma University Hospital [21], Fiche and Kuyu hospitals [16], Mozambique and Uganda [9], Tanzania [13]. This can be explained with children with advanced WHO clinical stage at initiation of ART could have an increased risk of or concomitant opportunistic infections which further impair the positive effects of the ART on immune cells.

In most studies both in our country and others show that low CD4 count at ART initiation and occurrence of severe opportunistic infection (like; tuberculosis and chronic diarrhea) were significant predictors of first-line ART treatment failure [9, 11, 13, 16, 21, 29], unlike the current study. This can be explained with, recently ART started for every HIV positive individuals and baseline CD4 is not done routinely for each and every patient, and severe opportunistic infections may not occur as like as the previous time [6].

Patients who had height for age in the third percentile or less at initiation of ART were found to have a higher probability of ART treatment failure [11] but in this study, due to the retrospective nature of the study and incomplete documentation of growth parameters, it was difficult to discuss on anthropometric assessment of study participants.

### Study limitations

Since the study was a retrospective study, it had its own limitation associated with poor documentation. Since the design is secondary, the study was unable to exhaustively explore all predictors variable that may have an effect on the treatment failure.

## Conclusions

In conclusion, the proportions of treatment failure among children on first-line ART at Northwest Amhara referral hospitals was found to be high and nearly half of the children experienced Virologic failure. Poor ART adherence, children without parents or died, WHO clinical stage IV at baseline and type of regimen patients took were found to be predictors of first-line ART treatment failure. Therefore, expanding access to routine viral load, CD4 and clinical monitoring is mandatory to detect and early intervene of treatment failures’ to improve outcomes for children on ART.

### Recommendations

Clinicians should provide targeted treatment approach with consideration of the risk of treatment failure (i.e., 1e) and proper documentation is necessary, since this study is depending on secondary data registered at each hospital, and the outcome relays on it. Expanding access to routine viral load, CD4 and clinical monitoring are mandatory to detect and early intervene of treatment failures’ to improve outcomes for children on ART. Patient caregivers or parents should strictly support children on medication adherence. Training to health professionals should be given time-based on revised guidelines, and follow up of treatment outcome should be monitored nationally to take the appropriate intervention.

## Supporting information

S1 File Raw data. (SAV)

## Abbreviations

AIDS: Acquired Immune Deficiency Syndrome
ART: Antiretroviral Therapy
HAART: Highly Active Antiretroviral Therapy
HIV: Human Immunodeficiency Virus
PMTCT: Prevention of Mother to Child Transmission
WHO: World Health Organization
