# Correction: Incidence and predictors of treatment failure among children on first-line antiretroviral therapy in Amhara Region Referral Hospitals, northwest Ethiopia 2018: A retrospective study

**DOI:** 10.1371/journal.pone.0217901

**Published:** 2019-06-04

**Authors:** Birtukan Aklog Yihun, Getiye Dejenu Kibret, Cheru Tesema Leshargie

There are errors in the author affiliations. The correct affiliations are as follows:

Birtukan Aklog Yihun^1^, Getiye Dejenu Kibret^2^, and Cheru Tesema Leshargie^2^

**1** Debre Markos Referral Hospital, Debre Marko, Ethiopia, **2** College of Health Science, Debre Markos University, Debre Marko, Ethiopia.
